# Anticholinergic burden and cognitive function in a large German cohort of hospitalized geriatric patients

**DOI:** 10.1371/journal.pone.0171353

**Published:** 2017-02-10

**Authors:** Barbara Pfistermeister, Thomas Tümena, Karl-Günter Gaßmann, Renke Maas, Martin F. Fromm

**Affiliations:** 1 Friedrich-Alexander-Universität Erlangen-Nürnberg, Institute of Experimental and Clinical Pharmacology and Toxicology, Fahrstraße 17, Erlangen, Germany; 2 GiB-DAT Database, Nürnberg, Germany; 3 Waldkrankenhaus St. Marien gGmbH, Internal Medicine III, Geriatrics Center Erlangen, Erlangen, Germany; University Of São Paulo, BRAZIL

## Abstract

**Purpose:**

Previous studies suggest an association between use of anticholinergic drugs in elderly patients and cognitive impairment. However, there are still limited data on the association of anticholinergic drug use and cognitive impairment as well as contribution of individual drugs to anticholinergic load using large, well-documented patient cohorts treated in geriatric units from Europe.

**Methods:**

We investigated 797,440 prescriptions to 89,579 hospitalized patients treated in geriatric units within the GiB-DAT database. Data of all patients discharged between 1 January 2013 and 30 June 2015 was included. The Anticholinergic Cognitive Burden (ACB) scale was used to classify anticholinergic drugs as definite (score 2 or 3) and possible anticholinergics (score 1). Cognitive function was determined using Mini-Mental State Examination (MMSE) and the standardized scale for dementia (4D+S).

**Results:**

In two multivariable logistic regression models age, sex, number of drugs and ACB total scores were identified as variables independently associated with cognitive impairment as measured by MMSE (odds ratio per ACB unit 1.114, 95% CI 1.099–1.130) or the diagnosis dementia (odds ratio 1.159 per ACB unit, 95% CI 1.144–1.173, both p < 0.0001). High anticholinergic load was associated with patients with severe cognitive impairment (p < 0.05 for all pairwise comparisons). ACB score 3 anticholinergic drugs contributed 77.9% to the cumulative amount of ACB points in patients with an anticholinergic load of 3 and higher.

**Conclusions:**

Using a cross-sectional study design, a significant positive association between anticholinergic drug load and cognitive impairment in European patients treated in specialised geriatric units was found. The most frequently used definitve anticholinergic drugs were quetiapine, amitriptyline and carbamazepine.

## Introduction

Anticholinergic drugs are commonly used for the treatment of various diseases. Drugs with therapeutic anticholinergic effects comprise e.g. antiemetics, anti-vertigo drugs, drugs for Parkinson’s disease and antispasmodics [1]. In addition, many commonly used drugs have anticholinergic side effects, e.g. antiarrhythmics, antihistamines, antidepressants and antipsychotics [1]. Known anticholinergic adverse effects last from dry mouth, constipation and visual impairment to confusion, delirium and cognitive decline [2].

Use of anticholinergic drugs in geriatric patients requires particular attention due to peripheral and central anticholinergic side effects [1, 3]. Due to multimorbidity and polypharmacy, they have a high probability of exposure to anticholinergic drugs and are especially vulnerable to side effects of anticholinergics [2–4]. It is well accepted that drugs with anticholinergic properties should be avoided as outline e.g. in the American Geriatrics Society Updated Beers Criteria, the STOPP/START criteria or the German PRISCUS list [5–7].

Cognitive impairment as a side effect of anticholinergic exposure has been described previously [8–12]. In a 2-year longitudinal study with 13,004 community-dwelling and institutionalized patients it was shown that the use of anticholinergics is associated with increases of the cumulative risk of cognitive impairment [11]. The longitudinal German Study on Aging, Cognition and Dementia in Primary Care Patients (AgeCoDe) showed an increased risk (HR = 2.081) for dementia by the chronic use of anticholinergics in a cohort of 2,605 patients [13]. Nevertheless it is still unknown, if patients profit from a reduction of anticholinergic load regarding cognitive function [14, 15].

It is important to note that the co-administration of several anticholinergics results in cumulative anticholinergic effects [16, 17]. For example, Mate et al. reported in a study of 1,044 community-dwelling elderly in a multivariate analysis that dementia (assessed by CAMCOG-R) was significantly associated with anticholinergic burden [16]. Use of medications with definite anticholinergics effects lead to a greater decline in the Mini-Mental State Examination (MMSE) of 0.33 points over two years compared to patients, which did not take definite anticholinergic drugs [11]. Moreover, it was shown in a prospective population-based cohort study in 3,434 participants that higher cumulative anticholinergic drug use is associated with an increased risk of dementia [12].

Several anticholinergic risk scales have been published, most of which use 4-point grading for the classification of the individual drugs [18, 19]. One frequently used classification to assess the overall anticholinergic load in patients is the Anticholinergic Cognitive Burden (ACB) scale, which classifies anticholinergic drugs in three categories [8, 20].

To the best of our knowledge, there are still limited data on the association of anticholinergic drug use and cognitive impairment from large, well-documented European patient cohorts. In particular, data are limited regarding the currently used spectrum of anticholinergic drugs in hospitalized geriatric patients. Therefore, we evaluated the epidemiology of anticholinergic burden and its association with cognitive impairment in a large sample of 89,579 hospitalized older patients in Germany. Moreover, in order to provide the basis for future prospective studies aiming at a reduction of the anticholinergic load, we report the most frequently used definite anticholinergic drugs in this cohort.

## Materials and methods

### Study setting and population

In 2000, the Geriatrics in Bavaria-Database (Geriatrie in Bayern Datenbank, GiB-DAT) was established as quality assurance project. Documentation is standardised in participating units. The conception, structure and results have previously been published [21–26].

More than 75 facilities with more than 100 geriatric units participate in the network covering about 91% of inpatient geriatric rehabilitation facilities and 55% of acute geriatric units in the state of Bavaria [27]. Approximately 50,000 data records are transferred to the central data base in an anonymized form per year [27]. So far the database includes more than 450,000 geriatric cases [27]. The present analyses of data provided by GiB-DAT were approved by the Ethics Committee of the Friedrich-Alexander-Universität Erlangen-Nürnberg (Erlangen, Germany).

For this retrospective cohort study, the data of all geriatric patients discharged between 1 January 2013 and 30 June 2015 with at least one drug at discharge were used. In total, 89,579 patients were included in this study. The following data is available in GiB-DAT and used for the present analysis: sociodemographic parameters (age, gender), duration of hospital stay, place of residence before admission and after discharge, Barthel score (a measure of performance in activities of daily living) at admission and before discharge, Timed Up and Go (TUG) test (a measure to assess a person's mobility) at admission and before discharge, and Geriatric Depression Scale (GDS) score at admission, number of diagnoses and group of diagnoses according to ICD-10 (main and subsidiary diagnoses were considered). Mini-Mental State Examination (MMSE) was performed at admission by the attending physician to measure cognitive impairment. The MMSE ranges from 0–30 (0–17 severe, 18–24 moderate, 25–30 no cognitive impairment) [28–30 and according to German adaptation]. If the patients had pathological MMSE values (i.e. MMSE < 25) and at least one of the following issues (general weakness, aphasia, depression, hemiparesis, hypacusis, nervousness, neuropsychological deficits, refusal), these MMSE values were not used for analyses as they are not meaningful due to the patients underlying condition.To assess severity of impairment of geriatric patients, the AFGIB (Ärztliche Arbeitsgemeinschaft zur Förderung der Geriatrie in Bayern) developed the 4D+S scale assessing dementia, depression, dysphagia and dysphasia as well as need for social action [23]. For this analysis, only the item dementia was used divided in no, mild, moderate and severe (for details please see S1 Table). The documentation of 4D+S was done during the course of the hospital stay. Drugs at discharge are documented with the appropriate Anatomical Therapeutic Chemical (ATC) classification system [31].

### Anticholinergics

To select drugs with anticholinergic effects, the 2012 update of the Anticholinergic Cognitive Burden (ACB) Scale was used [8, 20]. The ACB scale classifies anticholinergics in three groups: possible anticholinergics are listed with a score of 1 (e.g. aripiprazole, haloperidol or venlafaxine); definite anticholinergics are listed with a score of 2 (e.g. carbamazepine, pimozide) or 3 (e.g. amitriptyline, doxepin). Drugs with a score of 3 are associated with delirium. For numerical scoring, the score of each anticholinergic drug taken by the patient is summed up to the ACB total score. An ACB total score of 3 or higher is considered to be clinically relevant according to the ACB scale. The ACB scale was modified by omitting trospium (due to its very limited penetration into the central nervous system [32–34]) and by adding definite anticholinergic drugs (biperiden, metixen and maprotilin) with a score of 3. Patients with anticholinergics at discharge were identified by the respective ATC codes andACB total scores were calculated for the patients’ discharge medication.

### Statistical analysis

The data of the GiB-DAT-project were stored in MS Visual Fox Pro Database 9.0 and exported to SPSS ver. 20 (IBM, USA) for statistical analysis. Categorical data are presented as frequencies and percentages and continuous variables are presented as median and 25^th^-75^th^ percentile (interquartile range, IQR).

Kruskal-Wallis rank sum tests and subsequent Wilcoxon rank sum tests were performed with R (https://www.r-project.org) in order to analyze the association of ACB score with MMSE or 4D+S item dementia. To calculate adjusted odds ratios with 95% confidence invervals (CIs), an univariable and two multivariable logistic regression models (A and B) were performed. Model A used the MMSE score (dichotomized into no cognitive impairment vs. cognitive impairment) as dependent variable and using the age, sex, number of drugs and the total ACB score as covariates. Model B used the item dementia of the 4D+s scale (also dichotomized into no cognitive impairment vs. cognitive impairment) as dependent variable and using the age, sex, number of drugs and the total ACB score as covariates. Kendall’s Tau-b correlations were calculated to analyze correlations between ACB score and various variables (for details please see S2 Table). Mann-Whitney-U test was used for nonparametric comparison of MMSE scores in patients with antidementia drugsA value of p < 0.05 was considered to be statistically significant. The primary goal of this investigation was to test the null-hypothesis: There is no association of cognitive function measured by MMSE or 4D+S (item dementia) and ACB score.

## Results

### Patient characteristics

In total, data of 89,579 patients were included in the analysis. The median age was 82 years (25^th^– 75^th^ percentile: 77–87) and 66.3% of patients were female. They received a median of 9 (6–11) drugs. In total, 797,440 prescriptions were evaluated. 41,456 (46.3%) received at least one anticholinergic drug. Table 1 shows the characteristics of these patients.

**Table 1:** Patient characteristics (n = 41,456).

***Sociodemographic characteristics***	
Age (years)	82 (77–87)
Female sex (%)	67.1
***Clinical and functional status characteristics***	
Duration of hospital stay (days)	20 (15–23)
*Place of residence before admission (%)*	
Living alone	46.3
Privately living with others	43.5
Long-term care setting	10.2
Number of diagnoses	10 (8–14)
Barthel score (admission)	40 (25–60)
0030	70 (45–85)
Δ Barthel	20 (5–30)
*Timed up and go test (admission %)*	
Able to walk independently	49.8
Able to walk with assistant	29.7
Unable to walk	20.5
Timed up and go test (discharge %)	
Able to walk independently	73.4
Able to walk with assistant	18.7
Unable to walk	7.9
Mini-Mental State Examination (admission)	25 (20–27)
Geriatric depression scale (admission)	4 (3–7)
*Main and subsidiary diagnoses according to ICD 10 (%)*	
Circulatory system	87.9
Injury and poisoning	49.4
Symptoms, signs, and ill-defined conditions	56.0
Muscoloskeletal system and connective tissue	47.0
Nervous system and sense organs	35.4
Mental disorders	43.1
Others	41.2
Endocrine, nutritional and metabolic diseases, and immunity disorders	59.7
Genitourinary system	44.9
Respiratory system	29.1
Digestive system	27.6
Infectious and parasitic diseases	18.9
Neoplasms	11.3
Number of drugs per patient	10 (7–12)

Values are given as median (with interquartile range) or if indicated as percentages.

### Use of anticholinergic drugs

Of all patients receiving anticholinergic drugs, 30,828 (74.4%) received only one anticholinergic drug, 8,778 (21.2%) two, 1604 (3.9%) three and 246 (0.6%) more than three anticholinergic drugs, respectively. The mean ACB total score was 1.9 (1–12). 24,569 (59.3%) patients had an ACB total score of 1, 5,765 (13.9%) a score of 2 and 11,122 (26.8%) had a score of 3 or more.

**Fig 1:**
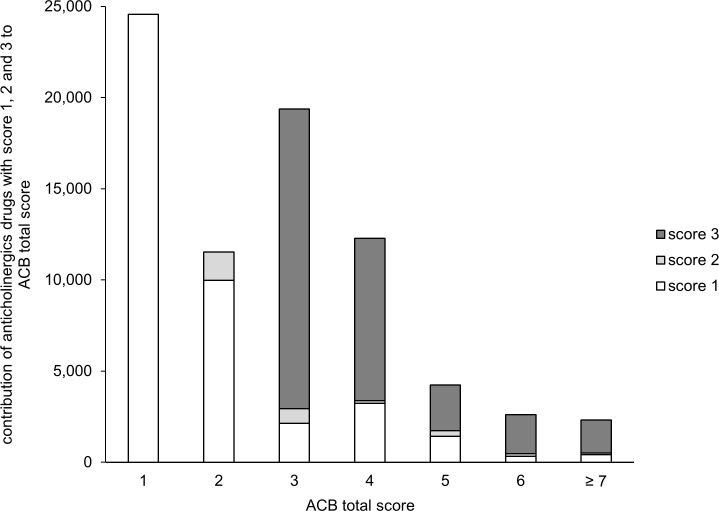
Contribution of drugs with an ACB score of 1, 2 or 3 to the cumulative ACB score of the entire study population. 54,211 anticholinergics were used with a cumulative ACB score of 76,934. *X-axis* groups of patients having individual ACB scores of 1, 2, 3, 4, 5, 6 or ≥ 7. *Y-axis* Cumulative ACB scores achieved in each group of patients and overview of ACB scoring points originating from drugs with ACB scores of 1,2 or 3, respectively.

Overall, 54,211 anticholinergics were used with a cumulative ACB score of 76,934. 42,087 (77.6%), 1,525 (2.8%) and 10,599 (19.6%) were anticholinergics with a score of 1, 2 and 3, respectively. In Fig 1, the contribution of anticholinergics with a score of 1, 2 or 3 to ACB total score is shown.

**Fig 2:**
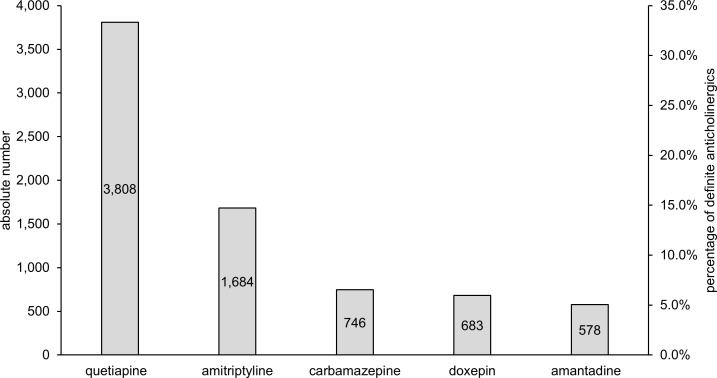
Most commonly used definite anticholinergic drugs (i.e. ACB score 2 or 3) among the total study population. *Left y-axis* Absolute number of drugs with definite anticholinergic properties received by patients. *Right y-axis* Proportion of the respective drug of all definite anticholinergics according to the ACB scale.

Fig 2 shows the 5 most commonly prescribed definite anticholinergics (i.e. ACB score 2 or 3). These 5 drugs represent 61.9% of all used definite anticholinergic drugs. The most common combinations of definite anticholinergics (i.e. both drugs with ACB score 2 or 3) were amantadine and quetiapine (59), amitriptyline and quetiapine (43) and amitriptyline and carbamazepine (36).

### Mutivariate analysis

For model A, 59,007 patient cases with complete data for all variables included into the model were available (S1 Fig). A logistic regression model was used to identify variables as independent predictors of cognitive impairment measured by the MMSE score (Table 2). Age, sex, number of drugs and ACB total scores were identified as variables independently associated with the MMSE score (p < 0.0001). ACB total score had an odds ratio of 1.114 per ACB unit (1.099–1.130, p < 0.0001). S3 Table shows in addition univariable analyses of various paratmeters with MMSE.

In model B, cognitive impairment was analysed by the item dementia of the 4D+S scale and 68,388 patient cases were available for the analysis (S1 Fig, Table 2). In accordance with model A, age, sex, number of drugs and ACB total score were identified as significant (p < 0.0001) variables associated with the item dementia of the 4D+S scale. ACB total score had an odds ratio of 1.159 per ACB unit (1.144–1.173, p < 0.0001).

**Table 2:** Multivariable analysis of factors associated with cognitive impairment.

	Odds ratio	95% CI	p value
***MMSE (model A)***			
Age (per year)	1.039	1.037–1.042	< 0.0001
Female sex	0.886	0.854–0.919	< 0.0001
Number of drugs (per drug)	0.971	0.966–0.976	< 0.0001
ACB total score (per ACB unit)	1.114	1.099–1.130	< 0.0001
***dementia (model B)***			
Age	1.042	1.040–1.045	< 0.0001
Female sex	0.794	0.767–0.820	< 0.0001
Number of drugs	0.965	0.961–0.970	< 0.0001
ACB total score (per ACB unit)	1.159	1.144–1.173	< 0.0001

Values are given as the odds ratio with the 95% confidence intervals (CI).

### Association with functional cognitive parameters

**Fig 3:**
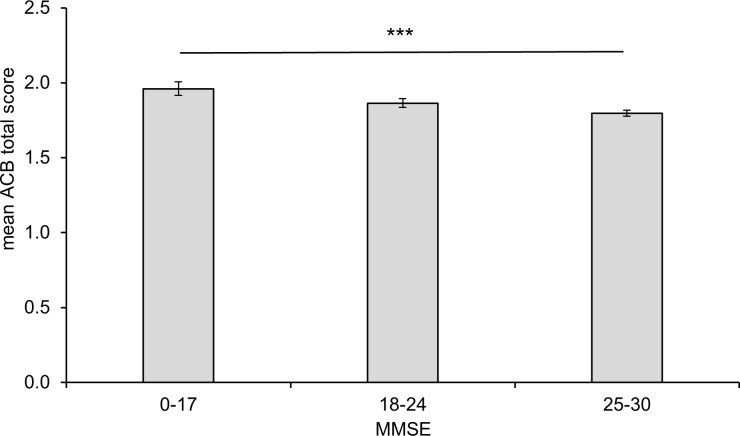
Cognitive impairment measured by the Mini-Mental State Examination (MMSE) and mean anticholinergic cognitive burden in patients of the GiB-DAT database. A MMSE score of 0–17 indicates severe, 18–24 moderate and 25–30 no cognitive impairment. Error bar 95% confidence interval, *** p < 0.001 for overall and all pairwise comparisons.

Fig 3 shows the association of the MMSE with the mean ACB total score. There is a significantly higher anticholinergic burden in patients with severe cognitive impairment compared with patients without cognitive impairment (p < 0.0001). The subsequent pairwise posthoc analysis was highly significant for all pairs (p < 0.001).

**Fig 4:**
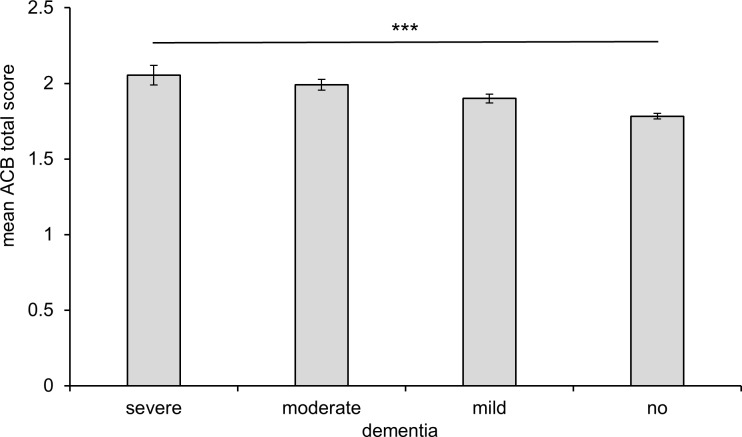
Cognitive impairment measured by the item dementia of the 4D+S scale and mean anticholinergic cognitive burden in patients of the GiB-DAT database. Error bar 95% confidence interval, *** p < 0.0001 for overall and pairwise comparisons except for the difference between moderate and severe dementia was borderline significance (p = 0.043).

Fig 4 highlights the association of the item dementia of the 4D+S scale with the mean ACB total score. Patients with severe dementia had a significantly higher ACB total score than patients without dementia (p < 0.0001). In line with the results for the MMSE, the subsequent pairwise posthoc analysis was highly significant (p < 0.0001) for all pairs with the exception of the difference between moderate and severe dementia (p = 0.043).

### Concomitant use of anticholinergic drugs with antidementia drugs

In our cohort, 5,812 patients received antidementia drugs (memantin, donepezil, galantamine, rivastigmine). 2,877 (6.9% of all patients with anticholinergics) used antidementia drugs and anticholinergics concomitantly. Mean ACB total score was 2.1 in this subgroup of patients. Comparing patients receiving antidementia drugs with or without anticholinergics concomitantly, the median MMSE is 20 in both groups (p = 0.651). In the group of patients, which received antidementia drugs, quetiapine was by far the most frequently used definitive anticholinergic drug (17.4% of the prescriptions of anticholinergic drugs in this subgroup), followed by amantadine (1.7%) and amitryptiline (1.5%).

## Discussion

In this retrospective cohort study, 46.3% of patients received at least one anticholinergic drug. Co-prescription of anticholinergics was present in one fourth of these patients and an according to the ACB score clinically relevant anticholinergic burden was also reached by one fourth of the patients receiving anticholinergic drugs [8]. The most commonly used definite anticholinergics in this cohort were quetiapine and amitriptyline.

Multivariable statistics revealed that age, sex, number of drugs and ACB total scores were associated with cognitive impairment categorized by both MMSE score and 4D+S scale. The anticholinergic burden was significantly higher in patients with severe cognitive impairment than in patients without cognitive impairment (mean ACB total score 2.0 vs. 1.8, determined with the MMSE (p < 0.0001)). These data are in line with the results from our data using the item ‘dementia’ of the 4D+S scale. Mean ACB score was highest in the group of patients with severe dementia, followed by lower ACB scores in the group of patients with moderate, mild or no dementia (p < 0.0001). These results support the suggestion that anticholinergic drug use is associated with cognitive impairment [2, 35, 36]. In the evaluation study of the ACB scale, the authors found a mean ACB score of 1.9 [8]. Fox et al. reported that 48% was taking anticholinergic drugs [11] and the mean total ACB score was 1.8 with a maximum of 12. In this study, it was not only shown that the use of anticholinergics is associated with greater risk of cognitive decline but also with a greater mortality over two years [11]. An association between anticholinergic drug exposure and cognitive impairment has frequently, but not always, been reported [37]. However, it is still not known, if the reduction of anticholinergic burden can improve cognitive function. In a small prospective study, Yeh et al. did not detect a significant effect on MMSE after reducing the anticholinergic burden within 12 weeks [15]. Similar results were published by Kersten et al., who observed in a small randomized controlled trial in 87 patients that there was no improvement of cognitive function, serum anticholinergic activity and mouth dryness in spite of a reduction of the anticholinergic burden in long-term residents within the 8 week study phase [14].

With regard to the use of anticholinergics, three-fourth of anticholinergic drugs used in the present study were anticholinergics with a score of 1. Already in 2001, Tune stated that the toxicity of anticholinergics is often the result of the cumulative anticholinergic burden rather than the effect of a single drug [2]. In a study with 1,044 participants, Mate et al. identified mild or potentially anticholinergics as major contributors to the anticholinergic load in people with dementia [16]. The cumulative use of anticholinergic and sedative drugs has also been associated with hospitalization and mortality [38]. In our cohort, we could show a significant association between a lower MMSE and the use of mild as well as definite anticholinergic drugs, but the odds ratio was higher for definite anticholinergic drug use (data not shown). These results support the assumption that also mild anticholinergic drugs have to be considered when reviewing a patient’s medication. On the other hand, it needs to be emphasized that in our study ACB score 3 anticholinergic drugs clearly most significantly contributed to the patients’ overall anticholinergic load for all patients having ACB total scores of 3 and higher (see Fig 1). ACB Score 3 drugs contributed 77.9% to the cumulative amount of ACB points in this group of patients, which is likely to suffer most from anticholinergic side effects.

## Limitations

We used the GiB-DAT database, a well-established, large database for quality assurance in Bavaria. Using a cross-sectional design, we found an association between the patients’ cognitive impairment measured by the Mini-Mental State Examination or the 4D+S item dementia with their anticholinergic burden. It is a limitation of our analysis that the MMSE is evaluated at admission while the medication is documented at discharge from the geriatric units in GiB-DAT. To confirm our results, we therefore used the diagnosis “dementia” of the 4D+S scale, which is used during the patients’ hospital stay. Since physicians in the geriatric units participating in GiB-DAT are specialized and well aware of problems arising from anticholinergic medications in elderly, we assume that the anticholinergic load might be lower at discharge compared to the time prior to admission and at the beginning of the hospital stay, thus underestimating the long-term anticholinergic load. Another point is that we can only show associations between the cognitive impairment of the patients and the anticholinergic burden, but not a causal relationship. Additionally, the length of intake of anticholinergic drugs is not known as no longitudinal data is available. Patients who develop anticholinergic side effects (such as decline in cognitive function attributed to anticholinergics) may stop taking the respective drugs (depletion of susceptible patients). Inclusion of all prevalent users, and not only those who recently started the drug could thus have distorted the study population (oversampling of patients at low risk for side effecs) and may have resulted in an underestimation of the observed effects. A "new user design" was not feasible in the present setting however. A further limitation is that repeated admissions of the same patients have been counted as separate cases, but the overall number of such cases is likely to be very small.

## Conclusions

In our study we could show for the first time a highly significant association of cognitive impairment (MMSE and dementia) with the anticholinergic burden, measured by the ACB score, in a large German geriatric cohort. Both ACB score 1 and score 3 drugs have a major contribution to overall anticholinergic burden. However, in patients with ACB scores of 3 and higher, which are probably the most relevant regarding anticholinergic side effects, clearly a relatively small number of class 3 drugs dominated regarding the anticholinergic load. Our data are a valuable basis for future randomized studies in larger patient groups to clarify if the reduction of the anticholinergic burden is beneficial for the patients’ cognitive function.

## Supporting information

S1 Fig Flow-chart for multivariable analyses. (TIF)

S1 Table Assessment of the item dementia of the 4D+S scale. (PDF)

S2 Table Correlation between ACB score and various variables. (PDF)

S3 Table Univariable analysis of the association of MMSE with various parameters. (PDF)
