# Supplementary figures and images for: Anticholinergic burden and cognitive function in a large German cohort of hospitalized geriatric patients
Source: PLoS One. 2017 Feb 10;12(2):e0171353. doi: 10.1371/journal.pone.0171353 (PMC5302450; doi:10.1371/journal.pone.0171353)

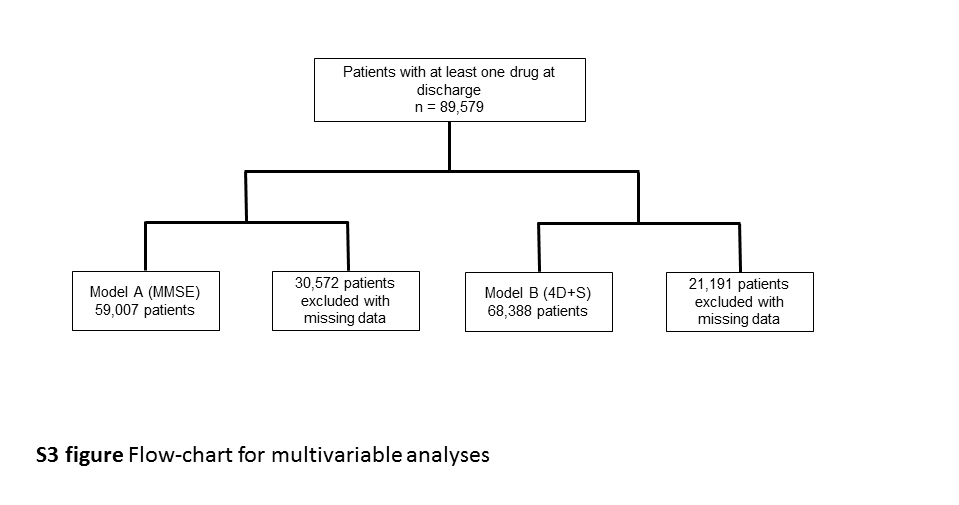

Supplement: S1 Fig — (TIF) [file pone.0171353.s001.tif]
