# Supplementary material for: Anticholinergic burden and cognitive function in a large German cohort of hospitalized geriatric patients
Source: PLoS One. 2017 Feb 10;12(2):e0171353. doi: 10.1371/journal.pone.0171353 (PMC5302450; doi:10.1371/journal.pone.0171353)
Supplement: S1 Table — (PDF) [file pone.0171353.s002.pdf]

S1 table Assessment of the item dementia of the 4D+S scale

Severity	***Description***
no dementia	- normal ageing<br>- forgetfulness
mild dementia	- fails at complex tasks in profession and society (e.g. journey to a new place)<br>- needs help with difficult taks of daily life (e.g. accounting, shopping, invitations)
moderate dementia	- needs help with selection of clothes and decision to bath<br>- needs help with dressing, bathing, toilet<br>- urinary incontinence and anal incontinence
severe dementia	- ability to speak 6 words<br>- cannot speak, sit, laugh<br>- cannot hold the head anymore
