# Supplementary material for: Anticholinergic burden and cognitive function in a large German cohort of hospitalized geriatric patients
Source: PLoS One. 2017 Feb 10;12(2):e0171353. doi: 10.1371/journal.pone.0171353 (PMC5302450; doi:10.1371/journal.pone.0171353)
Supplement: S2 Table — (PDF) [file pone.0171353.s003.pdf]

S2 table Correlation between ACB score and various variables

Variables	Kendall's Tau-b	p value
Age (years)	- 0.036	< 0.001
Sex	- 0.005	n.s.
Duration of hospital stay (days)	- 0.030	< 0.001
Barthel score (admission)	- 0.068	< 0.001
Δ Barthel	- 0.032	< 0.001
Mini-Mental State Examination (admission)	0.038	< 0.001
Number of drugs per patient	0.214	< 0.001
