# Supplementary material for: Anticholinergic burden and cognitive function in a large German cohort of hospitalized geriatric patients
Source: PLoS One. 2017 Feb 10;12(2):e0171353. doi: 10.1371/journal.pone.0171353 (PMC5302450; doi:10.1371/journal.pone.0171353)
Supplement: S3 Table — (PDF) [file pone.0171353.s004.pdf]

S3 table Univariable analysis of the association of MMSE with various parameters

Parameter	***Odds ratio***	***95 % CI***	***p value***
Age (years)	1.038	1.035 - 1.040	< 0.001
Female sex	0.988	0.953 – 1.023	n.s.
Number of drugs (per drug)	0.974	0.969 – 0.979	< 0.001
ACB total score (per ACB score unit)	1.070	1.056 – 1.084	< 0.001
Duration of hospital stay (days)	0.988	0.986 – 0.990	< 0.001
Barthel score (admission) (per Barthel score unit)	0.978	0.977 – 0.978	< 0.001
Barthel score (discharge) (per Barthel score unit)	0.974	0.973 – 0.975	< 0.001
Δ Barthel (per Barthel score unit)	0.993	0.992 – 0.994	< 0.001

n.s. not significant
